# The Clinical Features of Co-circulating Dengue Viruses and the Absence of Dengue Hemorrhagic Fever in Pakistan

**DOI:** 10.3389/fpubh.2020.00287

**Published:** 2020-06-17

**Authors:** Erum Khan, Dhani Prakoso, Kehkashan Imtiaz, Faisal Malik, Joveria Q. Farooqi, Maureen T. Long, Kelli L. Barr

**Affiliations:** ^1^Department of Pathology and Laboratory Medicine, Aga Khan University Hospital, Aga Khan University, Karachi, Pakistan; ^2^Department of Comparative Diagnostic and Population Medicine, College of Veterinary Medicine, University of Florida, Gainesville, FL, United States; ^3^Department of Biology, Baylor University, Waco, TX, United States

**Keywords:** dengue, co-infection, co-circulation, hemorrhagic fever, secondary infection

## Abstract

Dengue virus (DENV) is the most common and widespread arboviral infection worldwide. Though all four DENV serotypes cocirculate in nature, the clinicopathological framework of these serotypes is undefined in Pakistan. A cross-sectional, observational study was performed to document the circulation of various arboviruses in the Sindh region of Pakistan. Here we describe a population of patients diagnosed with DENV spanning a 2-year period. This study used an orthogonal system of NS1 antigen ELISA followed by RT-PCR for DENV detection and subtyping. A total of 168 NS1 positive patients were evaluated of which 91 patients were serotyped via RT-PCR. There was no significant difference between sex or age for infection risk and peak transmission occurred during the Autumn months. DENV2 was the most common serotype followed by DENV1 then DENV3, then DENV4. The data show that DENV1 patients were more likely to have abnormal liver function tests; DENV2 infected patients were more likely to exhibit arthralgia and neurological symptoms; DENV3 patients were more likely to complain of burning micturition and have elevated lymphocyte counts and low hematocrit; and DENV4 patients were more likely to report headaches and rash. Notably, no dengue hemorrhagic fever or other manifestations of severe dengue fever were present in patients with primary or secondary infections. We were able to identify significantly more NS1 antigen positive patients than RT-PCR. This study demonstrates that all four DENV serotypes are co-circulating and co-infecting in Pakistan.

## Introduction

Dengue virus (DENV) is the most common and widespread arboviral infection on Earth (1). DENV is a flavivirus composed of four antigenically distinct serotypes that causes a spectrum of disease ranging from mild febrile illness to death. Antibody-dependent enhancement during secondary DENV infections can exacerbate disease leading to dengue shock syndrome (DSS), dengue hemorrhagic fever (DHF), and dengue break-bone fever (2). Though these viruses occupy the same ecological niche, co-infections are rarely reported.

DENV is endemic to Pakistan along with West Nile virus (WNV), Japanese encephalitis virus (JEV), and Chikungunya virus (CHIKV) (3–5). These viruses have overlapping syndromes which complicates diagnosis leading to poorly defined epidemiology. Serological diagnosis is difficult due to antigenic cross reactivity of flaviviruses (6). Nucleic acid detection is equally as difficult due to the short duration of detectable viremia (7). Fortunately, advances in flaviviral diagnostics have significantly improved accurate and timely diagnosis which can have a direct and positive effect on patient care. For instance, several ELISA and lateral flow assays are commercially available that can simultaneously detect DENV antigen, IgM and IgG antibodies (8–11). This can help health care providers easily distinguish between early, primary, and secondary infections.

Though all four DENV serotypes cocirculate in nature, the clinical manifestations of these serotypes are undefined in Pakistan as well as the contribution of secondary and co-infections on the presentation of severe manifestations of disease. Here we describe a population of patients diagnosed with DENV over a 2-year period. Patients were divided according to their infecting serotype to document serotype-associated clinical features that might help differentiate one serotype from another in a primary health care setting.

## Methods

### Ethics Statement

Adult participants or parents/legal guardians of minors were enrolled under informed consent procedures which were reviewed and approved by the Ethics Review Committee at Aga Khan University (#3183-PAT-ERC-14) and the Institutional Review Board at the University of Florida (#201500908). All enrolled subjects gave written informed consent in accordance with the Declaration of Helsinki.

### Patient Samples

A cross-sectional, observational study was performed to document the circulation of various arboviruses in the Sindh region of Pakistan spanning 2015–2017 (4). Patients presenting with the CDC clinical description of arboviral disease including findings of rash, headache, arthralgia, myalgia, gastro-intestinal distress, acute hemorrhagic fever, acute flaccid paralysis, encephalitis, meningitis, and/or unexplained fever were recruited (4). Patients younger than 10 and older than 90 years of age were excluded. In total, 997 patients were enrolled, and clinical and demographic information was recorded. All enrolled patients provided a serum specimen which was screened for DENV NS1 antigen. 168 NS1 positive patients were evaluated for DENV serotype via RT-PCR.

### Case Definitions

All 4 DENV serotypes co-circulate in Pakistna and since secondary and co-infections can cause more severe forms of disease, it was necessary to classify RT-PCR + patients by their infection status. Primary infection was defined as NS1 AND RT-PCR positive but negative for neutralizing IgG by PRNT using patient specimen collected upon study enrollment. Secondary infection was defined as NS1 positive AND RT-PCR positive AND neutralizing IgG positive by PRNT using patient specimen collected upon study enrollment. Co-infections were defined as NS1 AND RT-PCR positive for 2 DENV serotypes but negative for neutralizing IgG by PRNT using patient specimen collected upon study enrollment.

### Serology

Primary DENV screening in patients was performed using a commercial ELISA (Panbio Dengue Early Rapid Test NS1 antigen capture test (Alere, Waltham, MA), following manufacturer's instructions. IgM ELISA was performed to rule out co-infection with WNV, JEV, CHIKV, and ZIKV with commercially purchased IgM ELISA assays including CHIKjj Detect, JE Detect, ZIKV Detect all from InBios International (Seattle, USA) per manufacturer's instructions. Plaque reduction neutralization testing (PRNT) was performed on all RT-PCR + patients as described elsewhere to determine if patients had previous DENV exposure (4, 12). Briefly, 100 infectious units (i.u.) of virus (calculated from stock virus titer) in PBS were incubated for 1 h at 37°C with 1:10 dilution of subject serum. Assay controls included 100 i.u. of virus in PBS, 100 i.u. of virus with 1:10 dilution of positive control serum, and a mock infected control consisting of PBS. Cells with inoculum were incubated at 37°C for 1 h after which the inoculum was removed and an overlay consisting of MEM with 10% FBS and 0.5% methylcellulose. Assays were incubated 3–7 days, depending on serotype, at 37°C after which monolayers were stained with Coomassie blue.

Viruses used included: DENV-1 (TS-SMAN), DENV-2 (NGC), DENV-3 (H87), and DENV-4 (H241). Viruses were obtained from BEI Resources and expanded once in Vero E6 cells. Patients were classified has having prior DENV exposure when there was neutralization of at least 80% at the 1:10 serum dilution. Specimens that neutralized more than 1 serotype were diluted out by 4-fold dilutions. The serotype with the most neutralization at the greatest dilution was identified as the infecting serotype.

### Nucleic Acid Tests

DENV primers and probes from the CDC RT-PCR assay were used to detect the NS5, envelope, or prM protein depending on serotype (4, 13). Briefly, RNA was isolated from human serum samples using a commercial viral RNA extraction kit (QIAamp viral RNA kit, Qiagen, Valencia, CA). RNA was reverse transcribed to cDNA (iScript cDNA Synthesis Kit, Bio-Rad, Hercules, California) and real-time PCR was performed using a commercial master mix (BioRad iTaq Universal Probes Supermix, BioRad, Hercules, CA, USA) and a commercial real-time PCR machine (BioRad CFX 96, BioRad) (4).

### Statistical Analysis

RT-PCR-positive patients were divided into 3 groups for statistical analysis which included primary infection, co-infection, and secondary infection per the case definitions above. We did not include patients with co-infections or secondary infections in the primary analysis because research has shown that these patients can have more severe manifestations of disease than patients with a primary infection with a single serotype (2, 14, 15). Clinical laboratory values were compared against the normal reference range for the appropriate sex as needed (i.e., hematocrit). Statistical analyses were performed on clinical data using MedCalc version 17.9.7 – 64-bit. Logistic regression for dichotomous independent variables were performed. Odds ratios were calculated with 95% confidence intervals. Ratios with a *p* < 0.05 were considered significant. When comparing serotypes with each other in respect to variables such as sex ratios, or age distribution, an ANOVA with Laverne's test, Tukey-Kramer *post-hoc* and Chi square for normal distribution was performed. When appropriate, pairwise comparisons were performed with a Student's *T*-test.

## Results

### Patient Characteristics

**Figure 1:**
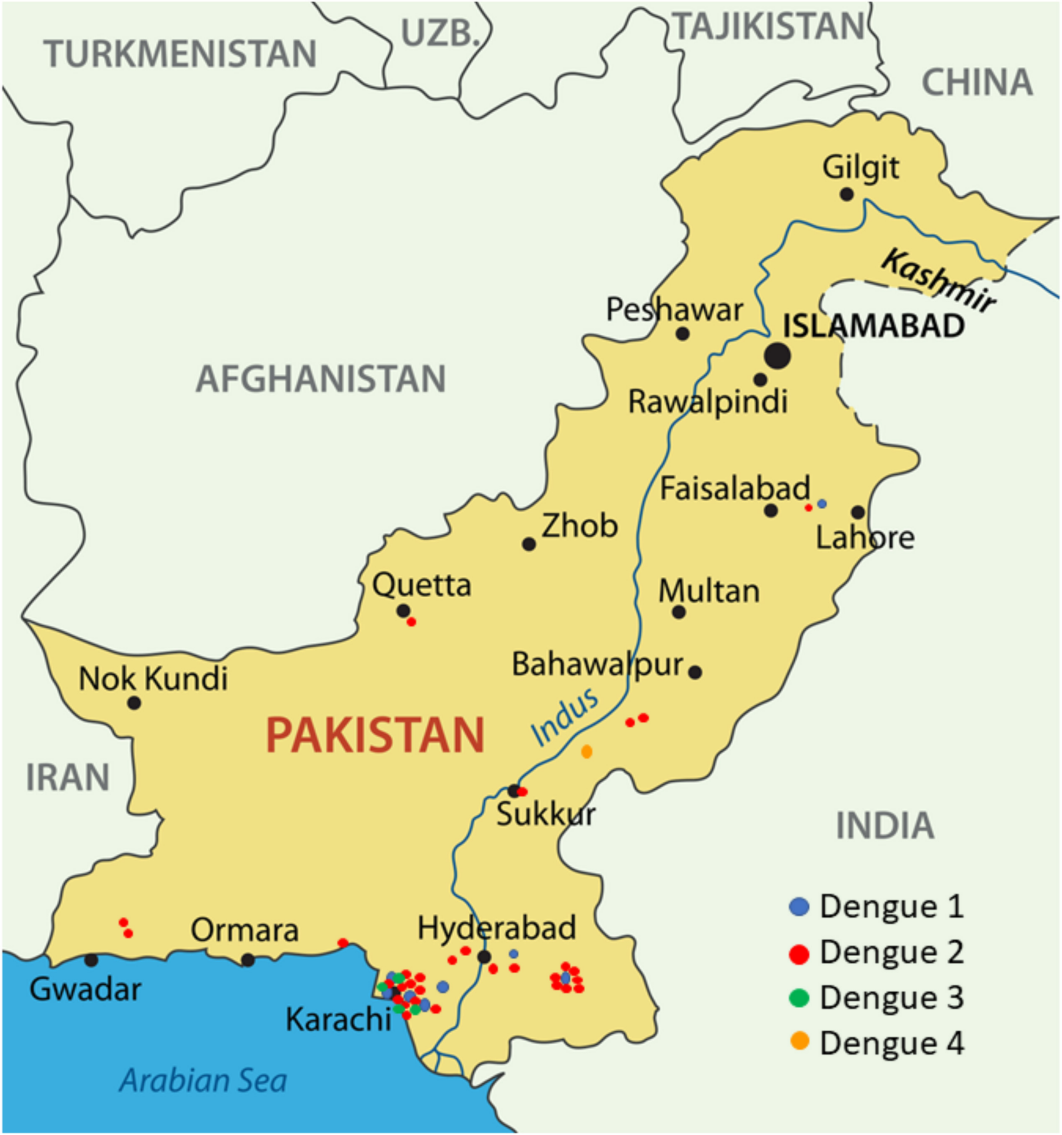
The locations of subjects infected with dengue virus during 2015–2017. The majority of cases were identified from Karachi.

**Figure 2:**
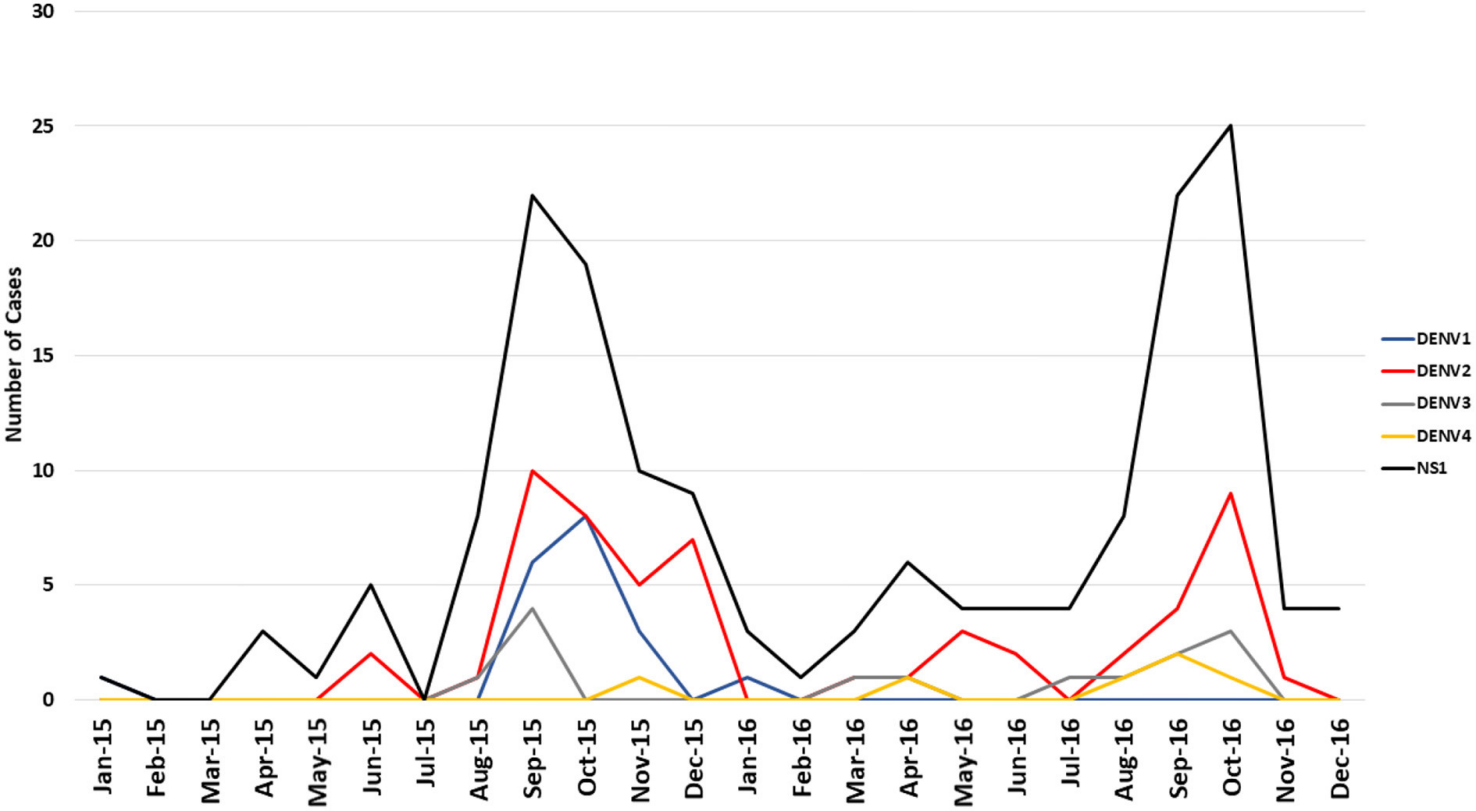
The sustained co-circulation of 4 dengue serotypes in the Sindh region of Pakistan 2015–2017. Data presented represents all RT-PCR positive samples including co-infections and secondary infections.

**Figure 3:**
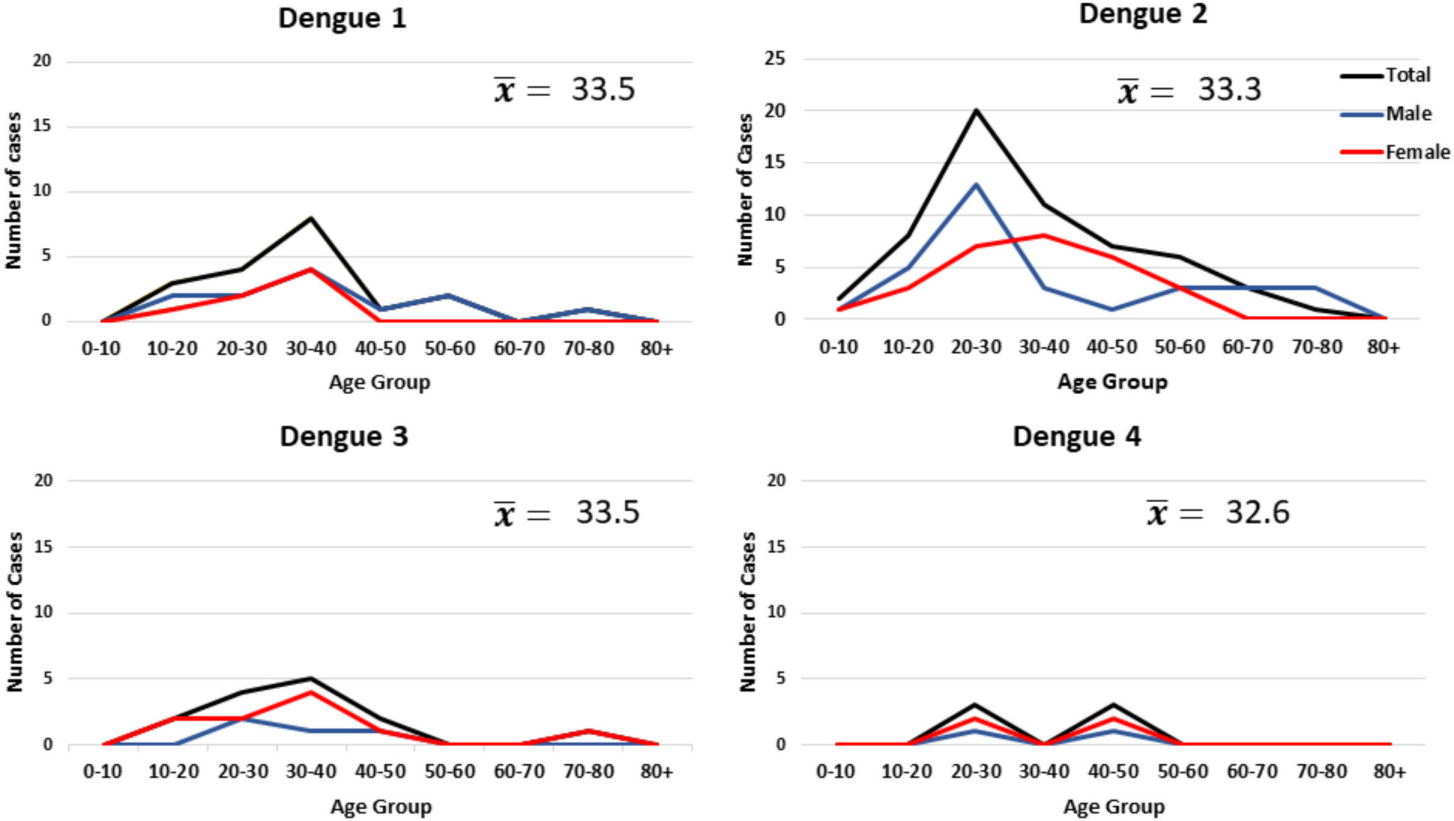
Age and gender of individuals infected with dengue virus. The data represents all primary and secondary infections.

A total of 168 NS1 positive patients were identified out of 997 subjects recruited from the Sindh province of Pakistan during 2015–2017. The vast majority of these 168 patients were recruited from Karachi, the most populous city in the region. Patients were also recruited from Mirpurkhas, Larkana, and Sukkur (Figure 1). A small number of patients were enrolled in Karachi from individuals visiting from other regions such as the Western province of Balochistan and the area around Lahore (Figure 1). These cases occurred throughout the year irrespective of holidays or festivals. All serotypes of DENV were identified year-round with the majority of cases identified during the autumn months (Figure 2). The average age of patients was 33 years and an equal proportion of males and females were observed (Figure 3). There was no significant difference between the serotypes for sex or age (Figure 3). Of the 168 NS1 positive patients, 91 were serotyped via RT-PCR. The remaining 77 patients were negative for all 4 DENV serotypes via RT-PCR and subtyping via plaque reduction neutralization test was performed. However, serological cross-reactivity prevented diagnosis thus, these patients were excluded from the analysis. DENV2 (*n* = 59) was the most commonly identified serotype by RT-PCR closely followed by DENV1 (*n* = 23). DENV3 was identified in 15 patients and DENV4 identified in 7 patients. All RT-PCR positive patients were also negative via IGM ELISA for JEV, WNV, CHIKV, and Zika viruses (data not shown).

Of the 23 DENV1 subtyped patients, 14 were RT-PCR positive for a second DENV serotype and 1 was identified as a secondary infection (Table S1). Of the 59 patients that were RT-PCR positive for DENV2, 12 had co-infections and 5 had secondary infections (Table S1). Of 12 patients RT-PCR positive for DENV 3, 2 were also positive for DENV 1 co-infections and 1 patient was positive for secondary DENV infection (Table S1). Seven individuals were positive for DENV4 via RT-PCR of which one patient was co-infected with DENV2 and another patient was positive for a secondary DENV infection (Table S1).

### Clinical Manifestations

Temperature, heart rate, and blood pressure readings were recorded upon enrollment. There were no significant manifestations for any of the serotypes (Table 1). Most patients from all DENV serotypes exhibited mean heart rates that were within the normal range of 60–100 beats per minute with no significant difference between serotypes (Table 1). Average blood pressure readings were within the normal range and <20% of patients (for all serotypes) exhibited abnormal readings. Average temperatures for DENV1 and DENV2 were 37.4 and 37.5°C, respectively (Table 1). Average temperatures for DENV3 and DENV4 were higher with average enrollment temperatures recorded at 38.0 and 38.2°C, respectively, with no significant difference between serotypes (Table 1).

**Table 1:** Vital statistics for patients with primary DENV infections.

	**Dengue 1**	**Dengue 2**	**Dengue 3**	**Dengue 4**
Heart rate (bpm)	98 (56–133)	94 (58–152)	97 (72–140)	100 (79–138)
Systolic (mmHg)	122 (92–160)	118 (88–176)	115 (100–134)	118 (105–122)
Diastolic (mmHg)	81 (55–112)	71 (32–92)	71 (56–83)	69 (54–84)
Temperature (°C)	37.4 (36.4–38)	37.5 (36–40.5)	38 (36.5–39)	38.2 (38–38.6)
Respirations (bpm)	20 (17-22)	20.3 (17-27)	21 (20–24)	20 (18–22)

*These patients were NS1 and RT–PCR positive for only one serotype and negative for DENV neutralizing antibodies via PRNT*.

We performed logistic regression to obtain odds ratios for a series of clinical hematological profiles commonly evaluated upon admission to hospital. Over 60% of patients infected with DENV1 exhibited elevated AST and ALT (Table 2). DENV1 patients were 2.0 times more likely to have elevated AST and 1.86 times more likely to have elevated ALT when compared with the other 3 serotypes (Table 2). Total lymphocyte counts were elevated in many patients however, DENV-3 infected individuals were 2.89 times more likely to exhibit elevated lymphocyte counts (Table 2). Thrombocytopenia is a common sign of DENV infection which was reported for the majority of patients for all serotypes though 100% of DENV1 and 80% of DENV4 infected patients exhibited low platelet counts and were at least 2.0 times more likely than any other serotype to have thrombocytopenia than the other serotypes (Table 2). Low hemoglobin counts were reported for 16% of DENV2 infected patients and 14% of DENV3 infected patients but not in DENV1 or DENV4 patients (Table 2). Below normal hematocrit levels were reported for 62% of DENV2 patients and 75% of DENV3 patients while no DENV1 and only 1 DENV4 infected patient had below normal hematocrit levels (Table 2). When compared with the other serotypes, DENV3 patients were twice as likely to exhibit low hematocrit levels (Table 2).

**Table 2:** Hematological profiles for patients with primary DENV infections.

	**Dengue 1**		**Dengue 2**		**Dengue 3**		**Dengue 4**	
	**Mean (range)**	**OR (95% CI)**	**Mean (range)**	**OR (95% CI)**	**Mean (range)**	**OR (95% CI)**	**Mean (range)**	**OR (95% CI)**
	***N* (%)**		***N* (%)**		***N* (%)**		***N* (%)**	
AST (IU/L)	228 (59–406)	2.0 (0.19 20.14)	139 (27-570)	0.75 (0.13–4.09)	114 (41–288)	0.83 (0.12–5.47)	Sample size too small	
	6 (86%)		12 (75%)		6 (50%)			
ALT (IU/L)	101 (20–247)	1.86 (0.38–8.98)	146 (16–751)	0.83 (0.25–2.72)	83.3 (28–277)	0.75 (0.17–3.29)	86.2 (28–221)	1.0 (0.12–7.81)
	5 (62%)		11 (48%)		4 (33%)		1 (16.6%)	
TLC (10^9^/L)	41 (12.9–52.7)	1.39 (0.3–6.41)	30 (4–55.5)	0.56 (0.2–1.56)	27.1 (8.2–48.1)	2.89 (0.7–11.87)	15.8 (3.5–24.1)	0.51 (0.07–3.27)
	5 (62%)		19 (46%)		9 (75%)		1 (20%)	
Platelets (10^9^/L)	46.5 (12-133)	**546**^**E+006**^	119 (19–330)	370^E+015^	134 (3–258)	0.94 (0.24–3.57)	114 (43–204)	2.0 (0.2–19.09)
	8 (100%)		23 (57%)		8 (67%)		4 (80%)	
Hemoglobin (gm/dL)	15.4 (12.2–17.2)	7.14^E−009^	13.5 (9.2–17.4)	1.81 (0.33–9.89)	13.5 (10.5–18.1)	1.43 (0.25–8.18)	13.8 (12.1–14.8)	7.14^E−009^
	0 (0%)		6 (16%)		2 (14%)		0 (0%)	
Hematocrit	44.8 (36–48.2)	0.10 0.009–1.09	39.2 (32.7–46.9)	1.04 (0.23–4.7)	37.5 (31.4–42.8)	2.0 (0.18–22.05)	40.2 (36.6–42.6)	629^E+006^
	0 (0%)		10 (62%)		3 (75%)		1 (25%)	

*These patients were NS1 and RT-PCR positive for only one serotype and negative for DENV neutralizing antibodies via PRNT. Bold font indicates statistical significance. OR, odds ratio; CI, confidence interval; N, number of patients*.

We performed logistic regression to obtain odds ratios for a series of common DENV-associated symptoms in order to determine if there were serotype-associated symptoms. Myalgia was a common symptom of all serotypes with at least 45% of patients reporting body aches (Table 3). Nausea and vomiting were also common complaints with at least 62% of all patients, for all serotypes, reporting these symptoms (Table 3). Eye pain was rarely reported with only 7% of DENV2 patients complaining of this symptom (Table 3). Overall, at least 58% of all patients were given a provisional diagnosis of dengue fever with 75% of DENV1 patients obtaining this diagnosis (Table 3). For patients without a provisional diagnosis of dengue fever, antibiotics were usually prescribed (*P* = 0.0007) (Table 3).

**Table 3:** Symptoms for patients with primary DENV infections.

	**Dengue 1**	**Dengue 2**	**Dengue 3**	**Dengue 4**
	**OR (95% CI)**	**OR (95% CI)**	**OR (95% CI)**	**OR (95% CI)**
	***N* (%)**	***N* (%)**	***N* (%)**	***N* (%)**
Arthralgia	0.0	4.0 (0.45–35.35)	2.34^E−009^	2.33 (0.22–24.4)
	0	6 (14.2%)	0	1 (20%)
Myalgia	1.11 (0.25–4.84)	0.76 (0.28–2.05)	1.11 (0.31–3.89)	1.71 (0.26–10.93)
	4 (50%)	19 (45.2%)	6 (50%)	3 (60%)
Headache	0.45 (0.06–2.91)	1.02 (0.37–2.8)	0.69 (0.15–3.09)	6.78 (0.71–64.42)
	2 (25%)	17 (40.4%)	4 (33.3%)	4 (80%)
Rash	0.74 (0.08–6.82)	0.59 (0.15–2.28)	1.10 (0.2–5.99)	4.25 (0.61–29.520
	1 (12.5%)	5 (12%)	2 (16.6%)	2 (40%)
Hemorrhagic manifestations	1.63 (0.31–10.75)	1.23 (0.33–4.61)	0.90 (0.17–4.76)	6.78^E−009^
	2 (25%)	8 (19%)	2 (16.6%)	0
Fever ≥38°C	**0.08 (0.01**–**0.75)**	1.50 (0.54–4.15)	0.92 (0.24–3.390	618^E+006^
	1 (12.5%)	23 (54.7%)	6 (50%)	5 (100%)
Nausea/vomiting	0.62 (0.13–2.89)	1.32 (0.44–3.92)	0.75 (0.19–2.86)	4.3 (0.23–78.63)
	5 (62.5%)	31 (73.8)	8 (66.6%)	4 (80%)
Burning micturition	0.79 (0.08–7.25)	0.54 (0.13–2.09	2.28 (0.49–10.53)	1.47 (0.14–14.72)
	1 (12.5%)	5 (12%)	3 (25%)	1 (20%)
Neurologic symptoms	2.37^E−009^	3.1 (0.61–15.88)	1.02 (0.19–5.47)	6.92^E−009^
	0	9 (21.4%)	2 (16.6%)	0
Dengue fever diagnosis	1.62 (0.27 to 9.49)	0.96 (0.33–2.8)	0.87 (0.22–3.38)	0.73 (0.08–6.58)
	6 (75%)	27 (62.4%)	7 (58.3%)	3 (60%)
Antibiotics prescribed	0.40 (0.07–2.15)	1.42 (0.50–4.03)	1.13 (0.3–4.2)	0.88 (0.13–5.670
	2 (25%)	18 (42.8%)	5 (41.6%)	2 (40%)
Eye pain	0.0	204^E+006^	6.88^E−009^	0.0
	0	3 (7%)	0	0
Fever ≥38.5°C	**1.83**^**E−009**^	0.91 (0.26–2.19)	2.78 (0.7–9.1)	1.55 (0.53–22.76)
	0	12 (26.2%)	6 (50%)	2 (40%)

*Logistic analysis was performed in which a single serotype was compared against the other serotypes. These patients were NS1 and RT-PCR positive for only one serotype and negative for DENV neutralizing antibodies via PRNT. Bold font indicates statistical significance. OR, odds ratio; CI, confidence interval; N, number of patients*.

The data indicates that patients with DENV1 infections were less likely to report dengue-associated symptoms that any other serotype. These patients had fewer complaints of arthralgia, myalgia, rash, nausea, and eye pain (Table 3). These patients were also significantly less likely to have a fever >38°C (*P* = 0.0261) (Table 3). Patients with DENV2 infections were 4.0 times more likely to complain of arthralgia than the other serotypes (Table 3). DENV2 patients were also 3.1 times more likely to exhibit neurological symptoms including, altered mental status, confusion, acute disseminated encephalomyelitis, or encephalitis (Table 3). Patients with DENV3 infections were unlikely to complain of arthralgia or other DENV-associated symptoms (Table 3). These patients were 2.28 times more likely to complain of burning micturition and 2.78 times more likely to have a fever >38.5°C than the other serotypes (Table 3). Nearly 80% of patients with DENV4 infection were most likely to exhibit dengue-like symptoms with patients being 6.78, 4.25, and 4.3 time more likely to report headache, rash, and nausea, respectively (Table 3).

### Co-infections

Co-infections of 2 different DENV serotypes were detected by RT-PCR in 14 individuals (Table S1). We performed logistic regression to obtain odds ratios, comparing these patients with all patients with primary infections. Research has shown that co-infections can produce more severe manifestations of disease (15). Thus, our goal was to determine if there were any clinical features that might be associated with infection with more than one DENV serotype. For our patients, 1 individual was co-infected with DENV serotypes 2 and 4. This patient had a fever of 39.4°C and hemorrhagic manifestations. This individual developed acute disseminated encephalomyelitis (ADEM). Three patients were co-infected with DENV serotypes 2 and 3 and 10 patients were co-infected with DENV1 and DENV2 (Table S1). These patients had relatively mild manifestations of dengue fever complaining of headache, myalgia, and nausea. Patients with co-infections were indistinguishable from patients with a single infection on the basis of their clinical manifestations (Table 4). Overall, patients with co-infections exhibited the same clinical profiles as patients infected with a single serotype (Table 4). Co-infected patients were significantly less likely to have thrombocytopenia or low hematocrit levels than patients infected with a single serotype (Table 4).

**Table 4:** Symptoms and hematological profiles for patients with DENV co-infections and secondary infections.

	**Co-infection**	**Secondary infection**
	**OR (95% CI)**	**OR (95% CI)**
	***N* (%)**	***N* (%)**
Arthralgia	2.85 (0.72–11.31)	1.42 (0.14–13.65)
	4 (26.6%)	1 (12.5%)
Myalgia	0.36 (0.1–1.24)	1.45 (0.3–7.02)
	4 (26.6%)	4 (50%)
Headache	0.88 (0.28–2.73)	3.70 (0.66–20.49)
	6 (40%)	5 (62.5%)
Rash	0.77 (0.15–3.92)	0.90 (0.09–8.3)
	2 (13.3%)	1 (12.5%)
Hemorrhagic manifestations	0.30 (0.036–2.54)	6.72^E−009^
	1 (6.6%)	0
Fever ≥38°C	0.77 (0.25–2.33)	1.03 (0.27–3.92)
	10 (71.4%)	7 (87.5%)
Nausea/vomiting	0.50 (0.16–1.56)	0.79 0.13–4.68
	8 (53.3%)	5 (62.5%)
Burning micturition	2.23^E−009^	6.97^E−009^
	0	1 (12.5%)
Neurologic symptoms	0.34 (0.04–2.84)	0.84 (0.09–7.76)
	1 (6.6%)	1 (12.5%)
Dengue fever diagnosis	1.12 (0.34–3.64)	3.07 (0.34–27.11)
	9 (60%)	5 (62.5%)
Antibiotics prescribed	1.71 (0.56–5.18)	1.3 (0.31–5.34)
	6 (40%)	0
Eye pain	6.81^E−009^	3.55 0.31–39.70
	0	1 (12.5%)
ALT	1.25 (0.29–5.280	5.0 (0.53–46.35)
	3 (20%)	2 (25%)
AST	**0.22 (0.06–0.83)**	2.21^E−010^
	5 (33.3%)	5 (62.5%)
Systolic	0.51 (0.05–4.41)	7.15^E−009^
	1 (6.6%)	0
Diastolic	0.0	**(25.60 1.96–333.56)**
	0	2 (25%)
Heart rate	2.62 (0.78–8.81)	1.68 0.34–8.24)
	7 (46.6%)	5 (62.5%)
Hemoglobin	2.83 (0.6–13.28)	3.31 (0.51–21.13)
	3 (20%)	1 (12.5%)
Hematocrit	**0.20 (0.04–0.91)**	1.03^E−009^
	3 (20%)	2 (25%)
PLT	**0.28 (0.09–0.89)**	0.35 (0.07–1.74)
	7 (46.6%)	6 (75%)
TLC	561^E+006^	0.80 (0.04–13.44)
	5 (33.3%)	2 (25%)

*Co-infected patients were NS1 and RT-PCR positive for at least 2 serotypes and negative for DENV neutralizing antibodies via PRNT. Patients with secondary infections were NS1 positive and positive for one serotype via RT-PCR and possessed DENV neutralizing antibodies as determined by PRNT. Logistic analysis was performed in which co-infected or secondary infections were compared against primary DENV infections. Bold font indicates statistical significance. OR, odds ratio; CI, confidence interval; N, number of patients*.

### Secondary Infections

We performed logistic regression to obtain odds ratios comparing individuals with secondary DENV infections with all individuals with primary infections. Our goal was to identify any significant clinical features that might be associated with secondary infections. Secondary DENV infection commonly result in more sever disease including DHF and DSS (2, 14–16). All RT-PCR positive serum was used for PRNT assay against the 3 respective RT-PCR negative serotypes. Secondary DENV infections were identified in 8 individuals by PRNT (Table S1). One patient PCR positive for DENV1 had high titers of neutralizing antibodies to DENV2. Five individuals with active DENV2 infections had high neutralizing titers against DENV1. One patient with an active DENV3 infection had high titers of neutralizing antibodies against DENV2. One individual with an active DENV4 infection had previous exposure to DENV1. None of the patients with secondary infection exhibited symptoms consistent with DHF or DSS in fact, these patients were indistinguishable from patents with primary infections of any serotype on the basis of their clinical manifestations (Tables 3, 4).

## Discussion

Dengue fever has been described in historical records since 922 A.D. as a self-limiting febrile illness punctuated by joint pain and rash (17, 18). However, it was not until 1966 that DENV emerged in Pakistan where it was reported to cause non-specific febrile illness accompanied by gastrointestinal symptoms (19). DENV was not reported again in Pakistan until 1994 in Karachi where DENV1 and DENV2 were identified during a major outbreak (20, 21). In 2005, DENV3 was identified during an outbreak of DHF in Karachi (22, 23). By 2008, all 4 serotypes had been identified in Pakistan (24).

We identified co-infections of two DENV serotypes which, according to the literature, should have resulted in more severe manifestations of disease (18, 25–27). Our data show that only a single co-infection of DENV2 and DENV4 caused severe disease which appears to agree with other reports from India (18). Patients co-infected with DENV1 and DENV2 were symptomatically indistinguishable from patients infected with a single serotype. This also agrees with other reports that DENV1 co-infections do not result in severe forms of dengue fever (18). The window of detectable viremia via RT-PCR is 3–5 days. While it is possible that these patients were infected on separate occasions over a short duration of time to be detected via RT-PCR. These patients would systemically and immunologically progress as a co-infection from a single inoculation. The scenario that these patients were infected by a co-infected mosquito is much more likely as co-infection of vectors has been reported for DENV in multiple lab and field studies (28–30).

A noteworthy finding was the absence of DHF or other manifestations of severe dengue fever in our patients with secondary infections and all but 1 of the co-infected. A recent review of DENV infection in India showed that the overall percentage of severe DENV infection was 28.9% of all cases (31). DHF and severe DENV are reported at rates of 0.5–1% globally (32). The presence of a DENV-resistance gene has been proposed since numerous reports document the absence of DHF and severe DENV in persons of African descent, especially populations in Haiti (33–36). However, these theories have never been substantiated and the mechanism for this phenomenon are unknown. The relative absence of DHF in Pakistan and wide-spread occurrence of DHF in India reflect studies of the Caribbean islands and raises the question if there is a genetic or immunological factor in the population of Pakistan that confers resistance to severe DENV (2, 34–36).

The role of T cells in viral hemorrhagic fevers has not been characterized and the protective or pathogenic contribution of T cells is unclear (37). However, the protective role of memory CD8 cells presenting cross-reactive epitopes has been shown to protect against secondary DENV infections in endemic areas (38, 39). In addition, sequential flaviviral infections can also confer cross-protection against primary or secondary DENV infections (40).

Another plausible explanation could be that persistent exposure to co-circulating viral pathogens (DENV1–4, WNV, JEV) has corrupted the normal immune defenses of this population. Memory CD8 T cells have been shown to respond to antigens, independent of T cell receptors to pathogens that cause inflammation (41–43). This allows memory CD8 cells to contribute to the innate immune response by detecting changes in cytokine production and eliciting a defensive response (44–46). However, during chronic viral infections, CD8 cells become exhausted and lose their self-renewal properties and over time, become depleted (41, 47, 48). Exhausted CD8 cells also lose their ability to produce interferon-γ when exposed to cognate antigens (49, 50). It is theorized that this loss of antigenic recognition could be immunopathogenic and contribute to sustained chronic viral infections (41). It could be that CD8 exhaustion if contributing to the lack of enhanced immune response to subsequent DENV infections as a result of their antigenic cross-reactivity.

Another explanation may lie in the antigenic cross-reactivity of the 4 serotypes. Antibody-dependent enhancement of infection from prior exposures is accepted as the primary cause of DHF, DSS, and death (2). However, recent work from Nicaragua has shown that DENV antibodies can offer protection or susceptibility to enhancement that is dependent on antibody titer (51). Our data show that all four DENV serotypes continuously circulate in Pakistan which could result in secondary infections that occur during times when antibodies might offer protection instead of enhancement. The same can be said for Haiti where all 4 serotypes continuously circulate (33). Clearly, more research is warranted in this area.

This study used an orthogonal system of NS1 antigen detection followed by RT-PCR for DENV detection and subtyping. We were able to identify significantly more NS1 positive patients than RT-PCR likely due to the use of serum as our RNA source. A growing body of research is showing that recovery of viral RNA persists for much longer in urine, saliva, whole blood, and plasma (52–54). Although we did not perform any phylogenetic analysis, prior work has shown that all four DENV serotypes circulating in Pakistan are descendant from India (55). Interestingly, DENV emerged in India during 1963, just 3 years prior to the Pakistan emergence in 1966 (25, 56).

This study demonstrates that all 4 DENV serotypes are co-circulating and co-infecting in Pakistan. The data show that each DENV serotype displayed at least one symptom at a higher frequency than the other serotypes though unfortunately, the incidence of specific symptoms was not discreet and was identified at least once for all serotypes. However, if one applies the clinical data to the system, one can generalize how each serotype presents in febrile patients in Pakistan. DENV1 had the greatest impact on the liver with higher frequency of abnormal liver function tests; DENV2 affected the central nervous system and joints; DENV3 affected the urinary system and blood; and DENV4 caused headaches and rash. The ability of these 4 serotypes to occupy the same ecological niche has puzzled scientists for decades. Perhaps the ability for specific serotypes to preferentially infect specific cells contributes to their persistence in the environment. These viruses provide an opportunity for future studies in DENV pathogenesis.

## Data Availability Statement

All datasets generated for this study are included in the article/Supplementary Material.

## Ethics Statement

The studies involving human participants were reviewed and approved by Ethics Review Committee at Aga Khan University (#3183-PAT-ERC-14) and the Institutional Review Board at the University of Florida (#201500908). Written informed consent to participate in this study was provided by the participants' legal guardian/next of kin.

## Author Contributions

KB, EK, and ML conceived and designed the experiments. KB, KI, DP, FM, JF, EK, and ML performed experiments and analyzed data. KB drafted the manuscript. All other authors edited and approved the text.

## Conflict of Interest

The authors declare that the research was conducted in the absence of any commercial or financial relationships that could be construed as a potential conflict of interest.

## Abbreviations

ALT: Alanine transaminase
AST: Aspartate transaminase
CHIKV: Chikungunya virus
DENV: Dengue virus
DHF: Dengue hemorrhagic fever
DSS: Dengue shock syndrome
JEV: Japanese encephalitis virus
PRNT: Plaque reduction neutralization test
WNV: West Nile virus
ZIKV: Zika virus.

## References

[1] Bos S Gadea G Despres P. Dengue: a growing threat requiring vaccine development for disease prevention. Pathog Glob Health. (2018) 112:294–305. 10.1080/20477724.2018.1514136 30213255 PMC6381545

[2] Guzman MG Alvarez M Halstead SB. Secondary infection as a risk factor for dengue hemorrhagic fever/dengue shock syndrome: an historical perspective and role of antibody-dependent enhancement of infection. Arch Virol. (2013) 158:1445–59. 10.1007/s00705-013-1645-3 23471635

[3] Khan E Barr KL Farooqi JQ Prakoso D Abbas A Khan ZY Human west nile virus disease outbreak in Pakistan, 2015-2016. Front Public Health. (2018) 6:20 10.3389/fpubh.2018.00020 29535994 PMC5835076

[4] Khan E Farooqi JQ Barr KL Prakoso D Nasir A Kanji A. Flaviviruses as a cause of undifferentiated fever in Sindh Province, Pakistan: a preliminary report. Front Public Health. (2016) 4:8. 10.3389/fpubh.2016.00008 26909342 PMC4754388

[5] Hayes CG Baqar S Ahmed T Chowdhry MA Reisen WK. West Nile virus in Pakistan. 1. Sero-epidemiological studies in Punjab Province. Trans R Soc Trop Med Hyg. (1982) 76:431–6. 10.1016/0035-9203(82)90130-4 6926759

[6] Patel B Longo P Miley MJ Montoya M Harris E de Silva AM. Dissecting the human serum antibody response to secondary dengue virus infections. PLoS Negl Trop Dis. (2017) 11:e0005554. 10.1371/journal.pntd.0005554 28505154 PMC5444852

[7] de Vasconcelos ZFM Azevedo RC Thompson N Gomes L Guida L Moreira MEL. Challenges for molecular and serological ZIKV infection confirmation. Child Nervous Syst. (2018) 34:79–84. 10.1007/s00381-017-3641-5 29110196

[8] Basile AJ Goodman C Horiuchi K Sloan A Johnson BW Kosoy O. Multi-laboratory comparison of three commercially available Zika IgM enzyme-linked immunosorbent assays. J Virol Methods. (2018) 260:26–33. 10.1016/j.jviromet.2018.06.018 29964076 PMC7176053

[9] Liu LT Dalipanda T Jagilly R Wang YH Lin PC Tsai CY. Comparison of two rapid diagnostic tests during a large dengue virus serotype 3 outbreak in the Solomon Islands in 2013. PLoS ONE. (2018) 13:e0202304. 10.1371/journal.pone.0202304 30096193 PMC6086442

[10] Murugananthan K Coonghe PAD Ketheesan N Noordeen F. Comparison of a rapid immuno-chromatography assay with a standard ELISA for the detection of IgM and IgG antibodies against dengue viruses. Virusdisease. (2018) 29:199–202. 10.1007/s13337-018-0440-x 29911153 PMC6003062

[11] Wu D Zhao LZ Wu YH Zhang H Zhang M Tan QQ. Comparison of Dengue viral nonstructural protein 1 antigen testing kits. Zhonghua Yu Fang Yi Xue Za Zhi. (2018) 52:141–4. 10.3760/cma.j.issn.0253-9624.2018.02.005 29429267

[12] Barr KL Schwarz ER Prakoso D Imtiaz K Pu R Morris JG Jr. Strain-dependent activity of zika virus and exposure history in serological diagnostics. Trop Med Infect Dis. (2020) 5:38. 10.3390/tropicalmed5010038 32138262 PMC7157670

[13] Santiago GA Vergne E Quiles Y Cosme J Vazquez J Medina JF. Analytical and clinical performance of the CDC real time RT-PCR assay for detection and typing of dengue virus. PLoS Negl Trop Dis. (2013) 7:e2311. 10.1371/journal.pntd.0002311 23875046 PMC3708876

[14] Dhanoa A Hassan SS Ngim CF Lau CF Chan TS Adnan NAA. Impact of dengue virus (DENV) co-infection on clinical manifestations, disease severity and laboratory parameters. BMC Infect Dis. (2016) 16:406. 10.1186/s12879-016-1731-8 27514512 PMC4982428

[15] Soo KM Khalid B Ching SM Chee HY. Meta-analysis of dengue severity during infection by different dengue virus serotypes in primary and secondary infections. PLoS ONE. (2016) 11:e0154760. 10.1371/journal.pone.0154760 27213782 PMC4877104

[16] Zaidi MB Cedillo-Barron L Gonzalez YAME Garcia-Cordero J Campos FD Namorado-Tonix K. Serological tests reveal significant cross-reactive human antibody responses to Zika and Dengue viruses in the Mexican population. Acta Trop. (2020) 201:105201. 10.1016/j.actatropica.2019.105201 31562846

[17] Gubler DJ. Dengue/dengue haemorrhagic fever: history and current status. Novartis Foundation Symp. (2006) 277:3–16; discussion: 22; 71–3; 251–3. 10.1002/0470058005.ch2 17319151

[18] Vaddadi K Gandikota C Jain PK Prasad VSV Venkataramana M. Co-circulation and co-infections of all dengue virus serotypes in Hyderabad, India 2014. Epidemiol Infect. (2017) 145:2563–74. 10.1017/S0950268817001479 28726595 PMC9148808

[19] Qureshi JA Notta NJ Salahuddin N Zaman V Khan JA. An epidemic of Dengue fever in Karachi–associated clinical manifestations. J Pak Med Assoc. (1997) 47:178–81. 9301157

[20] Russell PK Buescher EL McCown JM Ordonez J. Recovery of dengue viruses from patients during epidemics in Puerto Rico and East Pakistan. Am J Trop Med Hyg. (1966) 15:573–9. 10.4269/ajtmh.1966.15.573 4957424

[21] Chan YC Salahuddin NI Khan J Tan HC Seah CL Li J. Dengue haemorrhagic fever outbreak in Karachi, Pakistan, 1994. Trans R Soc Trop Med Hyg. (1995) 89:619–20. 10.1016/0035-9203(95)90412-3 8594672

[22] Jamil B Hasan R Zafar A Bewley K Chamberlain J Mioulet V. Dengue virus serotype 3, Karachi, Pakistan. Emerg Infect Dis. (2007) 13:182–3. 10.3201/eid1301.060376 17370547 PMC2725812

[23] Khan E Hasan R Mehraj V Nasir A Siddiqui J Hewson R. Co-circulations of two genotypes of dengue virus in 2006 out-break of dengue hemorrhagic fever in Karachi, Pakistan. J Clin Virol. (2008) 43:176–9. 10.1016/j.jcv.2008.06.003 18639489

[24] Humayoun MA Waseem T Jawa AA Hashmi MS Akram J. Multiple dengue serotypes and high frequency of dengue hemorrhagic fever at two tertiary care hospitals in Lahore during the 2008 dengue virus outbreak in Punjab, Pakistan. Int J Infect Dis. (2010) 14(Suppl 3):e54–9. 10.1016/j.ijid.2009.10.008 20171916

[25] Vinodkumar CS Kalapannavar NK Basavarajappa KG Sanjay D Gowli C Nadig NG. Episode of coexisting infections with multiple dengue virus serotypes in central Karnataka, India. J Infect Public Health. (2013) 6:302–6. 10.1016/j.jiph.2013.01.004 23806706

[26] Thomas L Verlaeten O Cabie A Kaidomar S Moravie V Martial J. Influence of the dengue serotype, previous dengue infection, and plasma viral load on clinical presentation and outcome during a dengue-2 and dengue-4 co-epidemic. Am J Trop Med Hyg. (2008) 78:990–8. 10.4269/ajtmh.2008.78.990 18541782

[27] Bharaj P Chahar HS Pandey A Diddi K Dar L Guleria R. Concurrent infections by all four dengue virus serotypes during an outbreak of dengue in 2006 in Delhi, India. Virol J. (2008) 5:1. 10.1186/1743-422X-5-1 18182120 PMC2253528

[28] Thavara U Siriyasatien P Tawatsin A Asavadachanukorn P Anantapreecha S Wongwanich R. Double infection of heteroserotypes of dengue viruses in field populations of Aedes aegypti and Aedes albopictus (Diptera: Culicidae) and serological features of dengue viruses found in patients in southern Thailand. Southeast Asian J Trop Med Public Health. (2006) 37:468–76. 17120966

[29] Pessanha JE Caiaffa WT Cecilio AB Iani FC Araujo SC Nascimento JC. Cocirculation of two dengue virus serotypes in individual and pooled samples of Aedes aegypti and Aedes albopictus larvae. Rev Soc Bras Med Trop. (2011) 44:103–5. 10.1590/S0037-86822011000100023 21340419

[30] Vazeille M Gaborit P Mousson L Girod R Failloux AB. Competitive advantage of a dengue 4 virus when co-infecting the mosquito Aedes aegypti with a dengue 1 virus. BMC Infect Dis. (2016) 16:318. 10.1186/s12879-016-1666-0 27390932 PMC4939008

[31] Ganeshkumar P Murhekar MV Poornima V Saravanakumar V Sukumaran K Anandaselvasankar A. Dengue infection in India: a systematic review and meta-analysis. PLoS Negl Trop Dis. (2018) 12:e0006618. 10.1371/journal.pntd.0006618 30011275 PMC6078327

[32] Sanyaolu A Okorie C Badaru O Adetona K Ahmed M Global epidemiology of dengue hemorrhagic fever: an update. J Hum Virol Retrovirol. (2017) 5:179–186. 10.15406/jhvrv.2017.05.00179

[33] Halstead SB Streit TG Lafontant JG Putvatana R Russell K Sun W. Haiti: absence of dengue hemorrhagic fever despite hyperendemic dengue virus transmission. Am J Trop Med Hyg. (2001) 65:180–3. 10.4269/ajtmh.2001.65.180 11561700

[34] Kouri GP Guzmán MG Bravo JR Triana C. Dengue haemorrhagic fever/dengue shock syndrome: lessons from the Cuban epidemic, 1981. Bull World Health Org. (1989) 67:375–80. 2805215 PMC2491263

[35] Guzmán MG Kouri GP Bravo J Soler M Vazquez S Morier L. Dengue hemorrhagic fever in Cuba, 1981: a retrospective seroepidemiologic study. Am J Trop Med Hyg. (1990) 42:179–84. 10.4269/ajtmh.1990.42.179 2316788

[36] Kourí G Guzmán MG Valdés L Carbonel I del Rosario D Vazquez S. Reemergence of dengue in Cuba: a 1997 epidemic in Santiago de Cuba. Emerg Infect Dis. (1998) 4:89–92. 10.3201/eid0401.980111 9454563 PMC2627664

[37] Perdomo-Celis F Salvato MS Medina-Moreno S Zapata JC. T-cell response to viral hemorrhagic fevers. Vaccines. (2019) 7:11. 10.3390/vaccines7010011 30678246 PMC6466054

[38] Malavige GN McGowan S Atukorale V Salimi M Peelawatta M Fernando N. Identification of serotype-specific T cell responses to highly conserved regions of the dengue viruses. Clin Exp Immunol. (2012) 168:215–23. 10.1111/j.1365-2249.2012.04566.x 22471283 PMC3390523

[39] Hatch S Endy TP Thomas S Mathew A Potts J Pazoles P. Intracellular cytokine production by dengue virus-specific T cells correlates with subclinical secondary infection. J Infect Dis. (2011) 203:1282–91. 10.1093/infdis/jir012 21335561 PMC3069729

[40] Saron WAA Rathore APS Ting L Ooi EE Low J Abraham SN. Flavivirus serocomplex cross-reactive immunity is protective by activating heterologous memory CD4 T cells. Sci Adv. (2018) 4:eaar4297. 10.1126/sciadv.aar4297 29978039 PMC6031378

[41] Ingram JT Yi JS Zajac AJ. Exhausted CD8 T cells downregulate the IL-18 receptor and become unresponsive to inflammatory cytokines and bacterial co-infections. PLoS Pathog. (2011) 7:e1002273. 10.1371/journal.ppat.1002273 21980291 PMC3182940

[42] Lertmemongkolchai G Cai G Hunter CA Bancroft GJ. Bystander activation of CD8+ T cells contributes to the rapid production of IFN-gamma in response to bacterial pathogens. J Immunol. (2001) 166:1097–105. 10.4049/jimmunol.166.2.1097 11145690

[43] Berg RE Forman J. The role of CD8 T cells in innate immunity and in antigen non-specific protection. Curr Opin Immunol. (2006) 18:338–43. 10.1016/j.coi.2006.03.010 16616476

[44] Cousens LP Peterson R Hsu S Dorner A Altman JD Ahmed R. Two roads diverged: interferon alpha/beta- and interleukin 12-mediated pathways in promoting T cell interferon gamma responses during viral infection. J Exp Med. (1999) 189:1315–28. 10.1084/jem.189.8.1315 10209048 PMC2193028

[45] Sareneva T Matikainen S Kurimoto M Julkunen I. Influenza A virus-induced IFN-alpha/beta and IL-18 synergistically enhance IFN-gamma gene expression in human T cells. J Immunol. (1998) 160:6032–8. 9637519

[46] Raué H-P Brien JD Hammarlund E Slifka MK. Activation of virus-specific CD8+ T cells by lipopolysaccharide-induced IL-12 and IL-18. J Immunol. (2004) 173:6873–81. 10.4049/jimmunol.173.11.6873 15557182

[47] Zajac AJ Blattman JN Murali-Krishna K Sourdive DJ Suresh M Altman JD. Viral immune evasion due to persistence of activated T cells without effector function. J Exp Med. (1998) 188:2205–13. 10.1084/jem.188.12.2205 9858507 PMC2212420

[48] Fuller MJ Khanolkar A Tebo AE Zajac AJ. Maintenance, loss, and resurgence of T cell responses during acute, protracted, and chronic viral infections. J Immunol. (2004) 172:4204–14. 10.4049/jimmunol.172.7.4204 15034033

[49] Wherry EJ Ha S-J Kaech SM Haining WN Sarkar S Kalia V. Molecular signature of CD8+ T cell exhaustion during chronic viral infection. Immunity. (2007) 27:670–84. 10.1016/j.immuni.2007.09.006 17950003

[50] Mackerness KJ Cox MA Lilly LM Weaver CT Harrington LE Zajac AJ. Pronounced virus-dependent activation drives exhaustion but sustains IFN-γ transcript levels. J Immunol. (2010) 185:3643–51. 10.4049/jimmunol.1000841 20720198 PMC2933304

[51] Katzelnick LC Gresh L Halloran ME Mercado JC Kuan G Gordon A. Antibody-dependent enhancement of severe dengue disease in humans. Science. (2017) 358:929–32. 10.1126/science.aan6836 29097492 PMC5858873

[52] Van den Bossche D Cnops L Van Esbroeck M. Recovery of dengue virus from urine samples by real-time RT-PCR. Eur J Clin Microbiol Infect Dis. (2015) 34:1361–7. 10.1007/s10096-015-2359-0 25794553

[53] Andries AC Duong V Ly S Cappelle J Kim KS Lorn Try P. Value of routine dengue diagnostic tests in urine and saliva specimens. PLoS Negl Trop Dis. (2015) 9:e0004100. 10.1371/journal.pntd.0004100 26406240 PMC4583371

[54] Klungthong C Gibbons RV Thaisomboonsuk B Nisalak A Kalayanarooj S Thirawuth V. Dengue virus detection using whole blood for reverse transcriptase PCR and virus isolation. J Clin Microbiol. (2007) 45:2480–5. 10.1128/JCM.00305-07 17522268 PMC1951229

[55] Koo C Nasir A Hapuarachchi HC Lee K-S Hasan Z Ng L-C. Evolution and heterogeneity of multiple serotypes of Dengue virus in Pakistan, 2006-2011. Virol J. (2013) 10:275-. 10.1186/1743-422X-10-275 24007412 PMC3844417

[56] Sarkar JK Pavri KM Chatterjee SN Chakravarty SK Anderson CR. Virological and serological studies of cases of haemorrhagic fever in Calcutta. Material collected by the Calcutta school of tropical medicine. Indian J Med Res. (1964) 52:684–91. 14195508

