# Supplementary material for: The Clinical Features of Co-circulating Dengue Viruses and the Absence of Dengue Hemorrhagic Fever in Pakistan
Source: Front Public Health. 2020 Jun 17;8:287. doi: 10.3389/fpubh.2020.00287 (PMC7311566; doi:10.3389/fpubh.2020.00287)
Supplement: Supplementary file 1 [file Table_1.DOCX]

Table S1. Serological profiles of DENV NS1 positive patients. All NS1 positive patients were subjected to RT-PCR for each DENV serotype. RT-PCR positive specimens were subjects to a PRNT for each DENV serotype. + indicates that the specimen was positive for a specific serotype by RT-PCR. The Secondary Infection Column lists the serotype of the primary exposure as determined by PRNT of 100% neutralization at the 1:10 serum dilution. ND= Not Done.

| Lab I.D | Secondary infection | DENV 1 | DENV 2 | DENV 3 | DENV 4 |
| --- | --- | --- | --- | --- | --- |
| H-009-15 | - | - | - | - | - |
| H-034-15 | ND | - | - | - | - |
| H-082-15 | ND | - | - | - | - |
| H-112-15 | ND | - | - | - | - |
| H-119-15 | - | - | + | - | + |
| H-127-15 | 2 | + | - | - | - |
| H-136-15 | - | - | + | - | - |
| H-175-16 | ND | - | - | - | - |
| H-180-16 | - | - | + | - | - |
| H-185-16 | - | - | + | - | - |
| K-005-15 | - | - | - | - | - |
| K-006-15 | ND | - | - | - | - |
| K-008-15 | ND | - | - | - | - |
| K-019-15 | ND | - | - | - | - |
| K-022-15 | - | - | + | - | - |
| K-031-15 | - | - | + | - | - |
| K-035-15 | ND | - | - | - | - |
| K-051-15 | ND | - | - | - | - |
| K-054-15 | - | - | - | + |  |
| K-056-15 | ND | - | - | - | - |
| K-061-15 | 1 | - | + | - | - |
| K-071-15 | ND | - | - | - | - |
| K-073-15 | ND | - | - | - | - |
| K-074-15 | ND | - | - | - | - |
| K-079-15 | - | - | + | - | - |
| K-080-15 | ND | - | - | - | - |
| K-084-15 | ND | - | - | - | - |
| K-086-15 | - | - | + | - | - |
| K-087-15 | - | - | - | + | - |
| K-090-15 | - | + | + | - | - |
| K-092-15 | - | - | + | - | - |
| K-095-15 | - | + | - | - | - |
| K-096-15 | ND | - | - | - | - |
| K-097-15 | ND | - | - | - | - |
| K-098-15 | - | + | + | - | - |
| K-099-18 | - | + | + | - | - |
| K-100-15 | ND | - | - | - | - |
| K-101-15 | - | + | - | - | - |
| K-104-15 | - | + | - | + | - |
| K-105-15 | - | + | - | + | - |
| K-111-15 | - | + | - | + | - |
| K-114-15 | - | + | - | - | - |
| K-117-15 | - | + | + | - | - |
| K-121-15 | - | + | + | - | - |
| K-122-15 | - | - | + | - | - |
| K-124-15 | ND | - | - | - | - |
| K-125-15 | - | + | + | - | - |
| K-131-15 | - | + | + | - | - |
| K-138-15 | - | - | - | + | - |
| K-139-15 | ND | - | - | - | - |
| K-140-15 | - | + | + | - | - |
| K-141-15 | - | + | + | - | - |
| K-148-15 | 2 | + | - | - | - |
| K-152-15 | ND | - | - | - | - |
| K-153-15 | ND | - | - | - | - |
| K-154-15 | ND | - | - | - | - |
| K-155-15 | ND | - | - | - | - |
| K-158-15 | 1 | - | + | - | - |
| K-160-15 | ND | - | - | - | - |
| K-161-15 | - | - | + | - | - |
| K-166-15 | - | - | + | - | - |
| K-170-15 | - | - | + | - | - |
| K-172-15 | - | - | + | - | - |
| K-179-15 | 1 |  | + | - | - |
| K-182-15 | - | + | - | - | - |
| K-186-15 | - | + | - | - | - |
| K-189-15 | - | - | + | - | - |
| K-190-15 | - | - | + | - | - |
| K-194-15 | - | - | + | - | - |
| K-197-15 | - | - | + | - | - |
| K-198-15 | ND | - | - | - | - |
| K-204-15 | - | - | + | - | - |
| K-205-15 | 1 | - | + | - | - |
| K-207-15 | ND | - | - | - | - |
| K-210-16 | - | + | - | - | - |
| K-212-16 | ND | - | - | - | - |
| K-213-16 | ND | - | - | - | - |
| K-214-16 | ND | - | - | - | - |
| K-216-16 | - | + | - | - | - |
| K-219-16 | 2 | - | - | + | - |
| K-220-16 | - | - | - | - | - |
| K-221-16 | 1 | - | + | - | - |
| K-222-16 | ND | - | - | - | - |
| K-225-16 | ND | - | - | - | - |
| K-226-16 | ND | - | - | - | - |
| K-228-16 | - | - | - | + | - |
| K-229-16 | - | - | - | - | + |
| K-230-16 | 1 | - | + | - | - |
| K-235-16 | - | - | + | - | - |
| K-236-16 | ND | - | - | - | - |
| K-239-16 | - | - | + | - | - |
| K-240-16 | 1 | - | + | - | - |
| K-241-16 | ND | - | - | - | - |
| K-243-16 | - | - | + | - | - |
| K-246-16 | - | - | + | - | - |
| K-248-16 | ND | - | - | - | - |
| K-250-15 | - | - | - | + | - |
| K-253-16 | - | - | - | + | - |
| K-257-16 | ND | - | - | - | - |
| K-262-16 | ND | - | - | - | - |
| K-265-16 | ND | - | - | - | - |
| K-266-16 | - | - | - | - | + |
| K-268-16 | - | - | - | - | + |
| K-270-16 | ND | - | - | - | - |
| K-271-16 | ND | - | - | - | - |
| K-276-16 | - | - | - | + | - |
| K-278-16 | ND | - | - | - | - |
| K-280-16 | ND | - | - | - | - |
| K-282-16 | ND | - | - | - | - |
| K-286-16 | - | - | - | - | + |
| K-288-16 | ND | - | - | - | - |
| K-289-16 | - | - | + | - | - |
| K-290-16 | ND | - | - | - | - |
| K-292-16 | ND | - | - | - | - |
| K-293-16 | - | - | + | - | - |
| K-294-16 | - | - | + | - | - |
| K-297-16 | - | - | - | + | - |
| K-298-16 | 1 | - | - | - | + |
| K-299-16 | ND | - | - | - | - |
| K-305-16 | ND | - | - | - | - |
| K-306-16 | - | - | - | + | - |
| K-307-16 | ND | - | - | - | - |
| K-308-16 | ND | - | - | - | - |
| K-311-16 | ND | - | - | - | - |
| K-312-16 | ND | - | - | - | - |
| K-313-16 | ND | - | - | - | - |
| K-316-16 | - | - | + | - | - |
| K-317-16 | - | - | + | - | - |
| K-318-16 | - | - | + | - | - |
| K319-16 | ND | - | - | - | - |
| K-320-16 | ND | - | - | - | - |
| K-326-16 | ND | - | - | - | - |
| K-329-16 | - | - | + | - | - |
| K-330-16 | - | - | + | - | - |
| K-331-16 | ND | - | - | - | - |
| K-333-16 | - | - | + | - | - |
| K-336-16 | - | - | + | - | - |
| K-338-16 | - | - | - | + | - |
| K-341-16 | ND | - | - | - | - |
| K-344-16 | ND | - | - | - | - |
| K-345-16 | - | - | + | - | - |
| K-346-16 | - | - | - | - | + |
| K-350-16 | ND | - | - | - | - |
| K-351-16 | - | - | - | + | - |
| K-353-16 | ND | - | - | - | - |
| K-355-16 | ND | - | - | - | - |
| K-356-16 | ND | - | - | - | - |
| K-357-16 | - | - | - | + | - |
| K-360-16 | - | - | - | - | - |
| K-367-16 | ND | - | - | - | - |
| K-380-16 | ND | - | - | - | - |
| K-382-16 | - | - | + | - | - |
| K-386-16 | - | - | + | - | - |
| K-387-16 | ND | - | - | - | - |
| K-393-16 | ND | - | - | - | - |
| K-402-17 | ND | - | - | - | - |
| K-427-17 | ND | - | - | - | - |
| K-911-16 | ND | - | - | - | - |
| L-006-15 | ND | - | - | - | - |
| M-037-15 | - | - | + | - | - |
| M-051-15 | - | - | + | - | - |
| M-052-15 | - | - | + | - | - |
| M-059-15 | - | + | + | - | - |
| M-076-15 | - | - | + | - | - |
| M-135-16 | - | - | + | - | - |
| S-085-16 | - | - | + | - | - |
| S-091-16 | - | - | - | - | + |
